# Active Demethylation of Non-CpG Moieties in Animals: A Neglected Research Area

**DOI:** 10.3390/ijms20246272

**Published:** 2019-12-12

**Authors:** Marco Lucarelli, Giampiero Ferraguti, Andrea Fuso

**Affiliations:** 1 Department of Experimental Medicine, Sapienza University of Rome, 00161 Rome, Italy; giampiero.ferraguti@uniroma1.it (G.F.); andrea.fuso@uniroma1.it (A.F.); 2 Pasteur Institute Cenci Bolognetti Foundation, 00161 Rome, Italy

**Keywords:** DNA methylation, non-CpG methylation, active demethylation, next generation sequencing

## Abstract

The functional role of cytosine methylation in the CpG moieties of DNA, is well established in several biological functions. The interplay between CpG methylation and hypomethylation is a well-known mechanism of modulation of gene expression. However, the role of non-CpG methylation and active dynamics of demethylation is not clearly recognized. Although some evidence exists of a role of active non-CpG demethylation in the fast dynamics of transcriptional activation in animals, few studies deal with this topic. At present, active demethylation of non-CpG moieties is a neglected research area, in spite of the promise of significant novelties.

DNA methylation is an epigenetic mark involved in several biological functions, mainly transcriptional modulation, but also differential promoter usage, transcription elongation, alternative splicing and silencing of genomic parasitic elements [1]. Strong evidence exists of multifaceted functional roles of cytosine methylation in CpG moieties of DNA, in particular when organized in zones at high CpG density (the so-called “CpG islands”). The mandatory need for a process of demethylation of methylated CpGs highlighted a first mechanism consisting in the loss of DNA CpG methylation patterns as a consequence of DNA replication. This kind of demethylation is usually called “passive demethylation”. Later, evidence was produced on the fast dynamics of demethylation, often extremely faster than the cell cycle of studied cells and usually called “active demethylation” [2,3]. Possible mechanisms of active demethylation have been discussed for a long time and, finally, some mechanisms have been proposed. At present, the most reliable mechanism for active demethylation is that the oxidation of methyl-cytosines (with hydroxymethylcytosine as intermediate) in the end results in the demethylation depending on an excision-repair mechanism mediated by TET proteins [4]. More recently vis-a-vis the golden era of CpG methylation, non-CpG methylation has also gained attention while remaining a debated topic. In particular, the dispute about the structural peculiarity of the methylation of a cytosine not being included in a symmetrical CpG dinucleotide has been resolved: evidence now exists of its functional role, especially in genes without CpG islands [3,5,6,7,8]. In particular, non-CpG methylation/demethylation interplay appears to be involved in transcriptional regulation of genes with promoter characterized by a low-density of CpG and/or in fast and active demethylation dynamics [2,3,4]. The CpG to non-CpG quantitative ratio dramatically varies between different organisms and tissues, as well as during development. As examples, non-CpG methylation accounts for 35% of total DNA methylation in the adult brain [9] and reaches over 50% during aging [10]. In oocytes, over 50% of non-CpG methylation has been found [11]. A high proportion of non-CpG methylation (up to 25%) has also been found in embryonic stem cells with, however, very low levels detected in other cell types [12,13,14].

The study of DNA methylation and demethylation, especially of non-CpG moieties, has for some time suffered the need for elaborated techniques that were not easily replicated [15,16]. In particular, the scarce attention paid to non-CpG methylation has reasons that tap into technical limitations and the consequent experimental routine. Before the advent of the bisulfite assay, for example, the most commonly used technique for studying DNA methylation was digestion by methylation-sensitive endonuclease followed by PCR, and the most commonly used endonuclease was HpaII, which recognizes CpG methylation on the CCGG sequence. A closer look to the results obtained with the isoschizomer MspI (that is insensitive to CpG but sensitive to CpC methylation of the CCGG target sites) and the use of endonuclease recognizing different sequences, however, revealed that non-CpG methylation could occur [3]. Nevertheless, HpaII remained the most frequently (and practically the only) endonuclease used in this assay. Interestingly, when the bisulfite assay was published, the authors of the technique described the characteristics of the primers to be used in the PCR reactions and disclosed that non-CpG methylation could be detected [17]. Nevertheless, from that time on, the experimental routine has favored the use of primers designed by software-assisted applications working under the principle that non-CpG cytosines were mainly unmethylated. As we have demonstrated, these primers are unable to detect non-CpG and can also underestimate CpG methylation [3,5,16]. The quantitative aspect of the proportion of methylation at each cytosine position has also been a hard task. With the advent of next generation sequencing (NGS) methodology a jump towards a new era in DNA methylation and demethylation studies was expected. In effect, the considerable number of NGS studies in the field of epigenetics in the last 10 years has shed new light on DNA modifications. The first mammalian whole-genome bisulfite sequencing [14] demonstrated high levels of non-CpG methylation in human embryonic stem cells. This was a break point, especially for non-CpG methylation which was no longer considered a possible bisulfite non-conversion artifact and was, after several other studies, finally accepted as a real epigenetic modification. However, due to the rooted technical and conceptual biases affecting the study of non-CpG methylation discussed above, most NGS-based applications, so far, also rely on the use of primers that underestimate non-CpG methylation or pass through the enrichment in CpG-rich sequences. Our suggestion is therefore to promote the application of NGS analysis based on fragmented genomic DNA with unbiased adaptors. Using this strategy, there is no reason not to detect non-CpG methylation too.

With the aim of contributing to the state-of-the-art knowledge of DNA methylation and demethylation studies in the NGS era, we setup a Pubmed search (accessed 12 November 2019) aimed at the selection of papers dealing with DNA methylation of CpG and non-CpG moieties, their demethylation dynamics, and their study using NGS approaches. Trying to focus on studies in animals, we excluded plants from this search. The search terms and their combinations are reported in Table 1, together with a more explanatory description of the expected results of the search. The results have been analyzed, and should be interpreted, as a comparison between different terms. As the search is applied similarly to all categories, the proportion of articles selected for each category represents a suitable sampling. This remains the case, even though the absolute number of articles could vary using different search strings.

As reported in Figure 1, a significant number of papers (ID#1, 68586) deal with the general topic of DNA methylation. However, only a limited fraction of these papers explicitly deal with the structural features of CpG (ID#2, 16857; 24.6%) and non-CpG (ID#3, 287; 0.4%) methylation. In addition to the striking lack of studies on non-CpG methylation, also the overall number of studies (ID#2 + ID#3, 16857 + 287; 25.0%) that takes into account at least the existence of distinct structural features of cytosines that can be methylated, is low. This fact is even more evident if we consider that the vast majority of studies that do not explicitly refer to the kind of DNA methylation studied deal with CpG methylation, simply disregarding non-CpG methylation. The number of studies dealing with DNA demethylation, without addressing the aspect of active or passive dynamics, despite being lower than those treating the more general topic of DNA methylation, is substantial (ID#4, 10160; 14.8% of overall DNA methylation studies). Only a very small proportion of these papers specifically addresses the involvement of non-CpG moieties in DNA demethylation (ID#6, 57; 0.6%) with respect to CpG demethylation (ID#5, 3035; 29.9%). In this case, what also appears surprising is the overall small proportion of studies that take into account at least possible differences between CpG and non-CpG demethylation (ID#5 + ID#6, 3035 + 57; 30.4%). A small number of papers directly address some possible different demethylation dynamics between passive (ID#7, 109; 1.1%) and active (ID#10, 1159; 11.4%) demethylation. Moreover, only very few studies specifically separate passive demethylation between CpG (ID#8, 27; representing 24.8% of studies on passive demethylation but only 0.3% of overall studies on DNA demethylation) and non-CpG (ID#9, 2; representing 1.8% of studies on passive demethylation but only 0.02% of overall studies on DNA demethylation). A similar trend can be seen if we divide active demethylation between CpG (ID#11, 319; representing 27.5% of studies on active demethylation but only 3.1% of overall studies on DNA demethylation) and non-CpG (ID#12, 7; representing 0.6% of studies on active demethylation but only 0.07% of overall studies on DNA demethylation). On the other hand, it should be noted that papers not explicitly focusing on the structural and/or dynamic features of DNA methylation and demethylation, usually deal with passive CpG demethylation. As expected, great attention is paid to DNA methylation and NGS (ID#13, 2089 papers), even taking into account that this number of papers refers to a shorter period (approximately the last 10 years) than those about DNA methylation in general (about 50 years). Even using NGS, CpG methylation is more studied (ID#14, 780; representing 37.3% of studies on DNA methylation and NGS), than non-CpG methylation (ID#15, 39; representing 1.9% of studies on DNA methylation and NGS). The overall number of NGS-based studies that takes into account that distinct structural features of cytosines that can be methylated exist (ID#14 + ID#15, 780 + 39; 39.2%) is definitely higher than those without an NGS approach. The proportion of DNA demethylation studies by NGS is similar to that found for the studies performed without NGS (ID#16, 224; 10.7% of overall studies on DNA methylation and NGS) with a greater interest for active (ID#22, 29; 12.9% of studies on DNA demethylation and NGS) than passive (ID#19, 2; 0.9% of studies on DNA demethylation and NGS) demethylation. There are a good number of NGS-based studies on CpG demethylation (ID#17, 102; 45.5% of studies on DNA demethylation and NGS), but only a few NGS-based studies about non-CpG demethylation (ID#18, 2; 0.9% of overall studies on DNA demethylation and NGS). However, the proportion of studies accounting for possible differences between CpG and non-CpG demethylation (ID#17 + ID#18, 102 + 2; 46.4% of studies on DNA demethylation and NGS) is higher than those without an NGS approach. Furthermore, a very limited number of studies based on NGS and distinguishing between CpG (ID#20, 0 studies) and non-CpG (ID#21, 1 study) passive demethylation could be selected. Some studies based on NGS dealing with active CpG demethylation exist (ID#23, 10; representing 34.5% of studies on active DNA demethylation and NGS), on the contrary to studies based on NGS and dealing with non-CpG active demethylation (ID#24, 1 study).

DNA methylation and demethylation are well-recognized and well-studied research areas, as demonstrated by the considerable number of published papers. NGS approaches have greatly improved the studies at both single-gene and whole-genome level. Comparing the two most critical dynamic and structural aspects, active demethylation appears to be more studied than non-CpG methylation and, especially at whole genome level, appears to have benefited most from the application of NGS technology. On the contrary, non-CpG methylation and its possible active dynamics of demethylation, appear poorly studied. This has happened despite intriguing preliminary results, recently achieved by NGS methodologies that have greatly improved these kinds of studies bringing them to a high-resolution level. Further improvement can be expected from the very recent development of antibodies that only recognize methylated cytosine in non-CpG dinucleotides (for example CpA) and by using unbiased bisulfite approaches at whole genome level [16,18,19]. Active demethylation of non-CpG moieties in animals seems to be, to date, a neglected research area that, however, could hide significant novelties just around the corner and, consequently, deserves more attention.

## Figures and Tables

**Figure 1:**
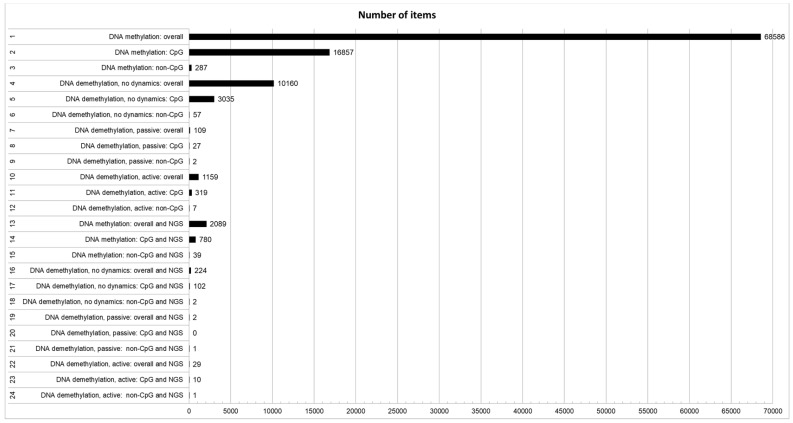
Number of published papers found in Pubmed according to the search terms used. The ID and the description of the search are reported in the vertical axis according to Table 1. The number of papers found are reported as data labels on the right of columns.

**Table 1:** Pubmed search terms used. In the first column the identification number of the search (ID) is reported. In second column the search expression is reported; when boolean operator is not indicated, the terms should be intended in AND. In the third column the description of the results of the search is reported; these descriptions are the same used in Figure 1.

ID.	Search	Description
1	(DNA methylation) NOT (plant OR plants)	DNA methylation: overall
2	((DNA methylation) AND ((CpG OR CG) NOT (non-CpG OR non-CG))) NOT (plant OR plants)	DNA methylation: CpG
3	((DNA methylation) AND (non-CpG OR non-CG)) NOT (plant OR plants)	DNA methylation: non-CpG
4	(DNA AND (demethylation OR hypomethylation)) NOT (plant OR plants)	DNA demethylation, no dynamics: overall
5	(DNA AND (demethylation OR hypomethylation) AND ((CpG OR CG) NOT (non-CpG OR non-CG))) NOT (plant OR plants)	DNA demethylation, no dynamics: CpG
6	(DNA AND (demethylation OR hypomethylation) AND (non-CpG OR non-CG)) NOT (plant OR pants)	DNA demethylation, no dynamics: non-CpG
7	(DNA AND ((passive demethylation) OR (passive hypomethylation))) NOT (plant OR plants)	DNA demethylation, passive: overall
8	(DNA AND ((passive demethylation) OR (passive hypomethylation)) AND ((CpG OR CG) NOT (non-CpG OR non-CG))) NOT (plant OR plants)	DNA demethylation, passive: CpG
9	(DNA AND ((passive demethylation) OR (passive hypomethylation)) AND (non-CpG OR non-CG)) NOT (plant OR plants)	DNA demethylation, passive: non-CpG
10	(DNA AND ((active demethylation) OR (active hypomethylation))) NOT (plant OR plants)	DNA demethylation, active: overall
11	(DNA AND ((active demethylation) OR (active hypomethylation)) AND ((CpG OR CG) NOT (non-CpG OR non-CG))) NOT (plant OR plants)	DNA demethylation, active: CpG
12	(DNA AND ((active demethylation) OR (active hypomethylation)) AND (non-CpG OR non-CG)) NOT (plant OR plants)	DNA demethylation, active: non-CpG
13	((DNA methylation) AND ((next generation sequencing) OR (parallel sequencing) OR WGBS OR (reduced representation) OR RRBS OR (restriction landmark genome scanning) OR RLGS OR (((post-bisulphite) OR (post-bisulfite) OR (post bisulphite) OR (post bisulfite)) AND (adapter OR adaptor) AND tagging) OR PBAT)) NOT (plant OR plants)	DNA methylation: overall and NGS
14	(((CpG OR CG) NOT (non-CpG OR non-CG)) AND (DNA methylation) AND ((next generation sequencing) OR (parallel sequencing) OR WGBS OR (reduced representation) OR RRBS OR (restriction landmark genome scanning) OR RLGS OR (((post-bisulphite) OR (post-bisulfite) OR (post bisulphite) OR (post bisulfite)) AND (adapter OR adaptor) AND tagging) OR PBAT)) NOT (plant OR plants)	DNA methylation: CpG and NGS
15	((non-CpG OR non-CG) AND (DNA methylation) AND ((next generation sequencing) OR (parallel sequencing) OR WGBS OR (reduced representation) OR RRBS OR (restriction landmark genome scanning) OR RLGS OR (((post-bisulphite) OR (post-bisulfite) OR (post bisulphite) OR (post bisulfite)) AND (adapter OR adaptor) AND tagging) OR PBAT)) NOT (plant OR plants)	DNA methylation: non-CpG and NGS
16	(DNA AND (demethylation OR hypomethylation) AND ((next generation sequencing) OR (parallel sequencing) OR WGBS OR (reduced representation) OR RRBS OR (restriction landmark genome scanning) OR RLGS OR (((post-bisulphite) OR (post-bisulfite) OR (post bisulphite) OR (post bisulfite)) AND (adapter OR adaptor) AND tagging) OR PBAT)) NOT (plant OR plants)	DNA demethylation, no dynamics: overall and NGS
17	(DNA AND (demethylation OR hypomethylation) AND ((CpG OR CG) NOT (non-CpG OR non-CG)) AND ((next generation sequencing) OR (parallel sequencing) OR WGBS OR (reduced representation) OR RRBS OR (restriction landmark genome scanning) OR RLGS OR (((post-bisulphite) OR (post-bisulfite) OR (post bisulphite) OR (post bisulfite)) AND (adapter OR adaptor) AND tagging) OR PBAT)) NOT (plant OR plants)	DNA demethylation, no dynamics: CpG and NGS
18	(DNA AND (demethylation OR hypomethylation) AND (non-CpG OR non-CG) AND ((next generation sequencing) OR (parallel sequencing) OR WGBS OR (reduced representation) OR RRBS OR (restriction landmark genome scanning) OR RLGS OR (((post-bisulphite) OR (post-bisulfite) OR (post bisulphite) OR (post bisulfite)) AND (adapter OR adaptor) AND tagging) OR PBAT)) NOT (plant OR plants)	DNA demethylation, no dynamics: non-CpG and NGS
19	(DNA AND ((passive demethylation) OR (passive hypomethylation)) AND ((next generation sequencing) OR (parallel sequencing) OR WGBS OR (reduced representation) OR RRBS OR (restriction landmark genome scanning) OR RLGS OR (((post-bisulphite) OR (post-bisulfite) OR (post bisulphite) OR (post bisulfite)) AND (adapter OR adaptor) AND tagging) OR PBAT)) NOT (plant OR plants)	DNA demethylation, passive: overall and NGS
20	(DNA AND ((passive demethylation) OR (passive hypomethylation)) AND ((CpG OR CG) NOT (non-CpG OR non-CG)) AND ((next generation sequencing) OR (parallel sequencing) OR WGBS OR (reduced representation) OR RRBS OR (restriction landmark genome scanning) OR RLGS OR (((post-bisulphite) OR (post-bisulfite) OR (post bisulphite) OR (post bisulfite)) AND (adapter OR adaptor) AND tagging) OR PBAT)) NOT (plant OR plants)	DNA demethylation, passive: CpG and NGS
21	(DNA AND ((passive demethylation) OR (passive hypomethylation)) AND (non-CpG OR non-CG) AND ((next generation sequencing) OR (parallel sequencing) OR WGBS OR (reduced representation) OR RRBS OR (restriction landmark genome scanning) OR RLGS OR (((post-bisulphite) OR (post-bisulfite) OR (post bisulphite) OR (post bisulfite)) AND (adapter OR adaptor) AND tagging) OR PBAT)) NOT (plant OR plants)	DNA demethylation, passive: non-CpG and NGS
22	(DNA AND ((active demethylation) OR (active hypomethylation)) AND ((next generation sequencing) OR (parallel sequencing) OR WGBS OR (reduced representation) OR RRBS OR (restriction landmark genome scanning) OR RLGS OR (((post-bisulphite) OR (post-bisulfite) OR (post bisulphite) OR (post bisulfite)) AND (adapter OR adaptor) AND tagging) OR PBAT)) NOT (plant OR plants)	DNA demethylation, active: overall and NGS
23	(DNA AND ((active demethylation) OR (active hypomethylation)) AND ((CpG OR CG) NOT (non-CpG OR non-CG)) AND ((next generation sequencing) OR (parallel sequencing) OR WGBS OR (reduced representation) OR RRBS OR (restriction landmark genome scanning) OR RLGS OR (((post-bisulphite) OR (post-bisulfite) OR (post bisulphite) OR (post bisulfite)) AND (adapter OR adaptor) AND tagging) OR PBAT)) NOT (plant OR plants)	DNA demethylation, active: CpG and NGS
24	(DNA AND ((active demethylation) OR (active hypomethylation)) AND (non-CpG OR non-CG) AND ((next generation sequencing) OR (parallel sequencing) OR WGBS OR (reduced representation) OR RRBS OR (restriction landmark genome scanning) OR RLGS OR (((post-bisulphite) OR (post-bisulfite) OR (post bisulphite) OR (post bisulfite)) AND (adapter OR adaptor) AND tagging) OR PBAT)) NOT (plant OR plants)	DNA demethylation, active: non-CpG and NGS
